# 10 practical priorities to prevent and manage serious allergic reactions: GA^2^LEN ANACare and EFA Anaphylaxis Manifesto

**DOI:** 10.1002/clt2.70009

**Published:** 2024-11-29

**Authors:** Antonella Muraro, Debra de Silva, Marcia Podesta, Aikaterini Anagnostou, Victoria Cardona, Susanne Halken, Pete Smith, Luciana Kase Tanno, Paul Turner, Margitta Worm, Montserrat Alvaro‐Lozano, Stefania Arasi, Anna Asarnoj, Simona Barni, Kirsten Beyer, Lucy A. Bilaver, Andrew Bird, Roberta Bonaguro, Helen A. Brough, R. Sharon Chinthrajah, Emma E. Cook, Céline Demoulin, Antoine Deschildre, Timothy E. Dribin, Motohiro Ebisawa, Montserrat Fernandez‐Rivas, Alessandro Fiocchi, David M. Fleischer, Eleanor Garrow, Jennifer Gerdts, Mattia Giovannini, Kirsi M. Järvinen, Mary Kelly, Edward F. Knol, Gideon Lack, Francesca Lazzarotto, Thuy‐My Le, Stephanie Leonard, Jay Lieberman, Michael Makris, Lianne Mandelbaum, Mary Jane Marchisotto, Gustavo Andres Marino, Francesca Mori, Caroline Nilsson, Anna Nowak‐Wegrzyn, Mikaela Odemyr, H. N. G. Oude Elberink, Kati Palosuo, Nandinee Patel, Jennifer Pier, Sung Poblete, Rima Rachid, Pablo Rodríguez del Río, Maria Said, Hugh A. Sampson, Angel Sánchez Sanz, Sabine Schnadt, Fallon Schultz, Alice Toniolo, Julia E. M. Upton, Carina Venter, Brian P. Vickery, Berber Vlieg‐Boerstra, Julie Wang, Graham Roberts, Torsten Zuberbier, GALEN ANACare Centres and EFA

**Affiliations:** ^1^ Padua University Hospital Padua Italy; ^2^ The Evidence Centre London UK; ^3^ European Federation of Allergy and Airways Diseases Patients' Associations Brussels Belgium; ^4^ Children's Hospital Houston Texas USA; ^5^ Baylor College of Medicine Houston Texas USA; ^6^ Hospital Universitari Vall d'Hebron Barcelona Spain; ^7^ Hans Christian Andersen Children's Hospital and University of Southern Denmark Odense Denmark; ^8^ Griffith University Southport Queensland Australia; ^9^ Hôpital Arnaud de Villeneuve ‐ University Hospital of Montpellier Montpellier France; ^10^ Imperial College London London UK; ^11^ Charite Univeritätsmedizin Berlin Berlin Germany; ^12^ Hospital Sant Joan de Déu Barcelona Spain; ^13^ Bambino Gesù Children's Hospital Rome Italy; ^14^ Astrid Lindgren Children's Hospital Karolinska University Hospital Stockholm Sweden; ^15^ Meyer Children's Hospital IRCCS Florence Italy; ^16^ German Center for Child and Adolescent Health Partner Site Berlin Berlin Germany; ^17^ Northwestern University Evanston Illinois USA; ^18^ University of Texas Southwestern University Dallas Texas USA; ^19^ Guy's and St Thomas' NHS Foundation Trust and King's College London London UK; ^20^ Stanford University Stanford California USA; ^21^ ATOPICCO Network for Children of the Earth Tokyo Japan; ^22^ AFPRAL Paris France; ^23^ Université de Lille Lille France; ^24^ Cincinnati Children's Hospital Medical Center and University of Cincinnati Cincinnati Ohio USA; ^25^ NHO Sagamihara National Hospital Sagamihara Japan; ^26^ Hospital Clinico San Carlos Universidad Complutense IdISSC Madrid Spain; ^27^ Pediatric Hospital Bambino Gesù Rome Italy; ^28^ University of Colorado and Children's Hospital Colorado Aurora Colorado USA; ^29^ Food Allergy and Anaphylaxis Connection Team West Chester Ohio USA; ^30^ Food Allergy Canada North York Ontario Canada; ^31^ University of Florence Florence Italy; ^32^ University of Rochester New York New York USA; ^33^ Anaphylaxis UK Farnborough UK; ^34^ University Medical Center Utrecht Utrecht The Netherlands; ^35^ University Utrecht Utrecht The Netherlands; ^36^ University of California, San Diego La Jolla California USA; ^37^ University of Tennessee Health Science Center and LeBonheur Children's Hospital Memphis Tennessee USA; ^38^ National and Kapodistrian University of Athens Athens Greece; ^39^ No Nut Traveler Inc Livingston New Jersey USA; ^40^ MJM Advisory LLC New York New York USA; ^41^ Hospital Universitario Austral and Fundación SOS Alergia Pilar Argentina; ^42^ Karolinska Institutet Stockholm Sweden; ^43^ Sachs' Children and Youth Hospital Stockholm Sweden; ^44^ New York University New York New York USA; ^45^ University of Warmia and Mazury Olsztyn Poland; ^46^ Astma‐ och Allergiförbundet Stockholm Sweden; ^47^ University of Groningen Groningen The Netherlands; ^48^ Helsinki University Hospital Helsinki Finland; ^49^ University of Rochester Rochester New York USA; ^50^ Food Allergy Research & Education (FARE) McLean Virginia USA; ^51^ Boston Children's Hospital Harvard Medical School Boston Massachusetts USA; ^52^ Hospital Infantil Universitario Niño Jesús Madrid Spain; ^53^ Allergy & Anaphylaxis Australia Sydney New South Wales Australia; ^54^ Icahn School of Medicine at Mount Sinai New York New York USA; ^55^ AEPNAA Spanish Association of People with Food and Latex Allergy Madrid Spain; ^56^ Deutscher Allergie‐ und Asthmabund Berlin North Rhine‐Westphalia Germany; ^57^ International FPIES Association Quincy Massachusetts USA; ^58^ University of Toronto Toronto Ontario Canada; ^59^ University of Colorado Denver Colorado USA; ^60^ Emory University School of Medicine and Children's Healthcare of Atlanta Atlanta Georgia USA; ^61^ OLVG Hospital Amsterdam The Netherlands; ^62^ University of Southampton Southampton UK; ^63^ St Mary's Hospital Southampton UK; ^64^ NIHR Biomedical Research Centre Southampton UK; ^65^ University Hospital Southampton NHS Foundation Trust Southampton UK; ^66^ Fraunhofer Institute for Translational Medicine and Pharmacology ITMP Immunology and Allergology Berlin Germany

**Keywords:** adrenaline, allergy, anaphylaxis, hypersensitivity

## Abstract

This Anaphylaxis Manifesto calls on communities to prioritise 10 practical actions to improve the lives of people at risk of serious allergic reactions. The Global Allergy and Asthma European Network and the European Federation of Allergy and Airways Diseases Patients' Associations (EFA) compiled patient‐centric priorities. We used qualitative consensus methods, research evidence and feedback from over 200 patient groups, stakeholder organisations and healthcare professionals. We encourage healthcare, education and food organisations to collaborate with people at risk of serious allergic reactions to tackle safety, anxiety and financial burdens for individuals and societies. Key priorities for prevention include awareness‐raising campaigns for the public and professionals, school and workplace initiatives and mandatory precautionary allergen labels on food. Priorities for improving immediate and long‐term management include educating healthcare professionals, patients and schools about when and how to use adrenaline, funding two approved adrenaline devices for everyone at risk, and facilitating access to allergy specialists. Integrated care pathways should include clinical and non‐clinical management options such as individualised risk assessment and quality of life assessment, self‐management plans, dietetic and psychosocial support and peer support. Organisations around the world are committing to work together towards these priorities.

## 1 WHY IDENTIFY PRIORITIES?

Anaphylaxis is a serious hypersensitivity reaction. Hypersensitivity means that the body's immune system reacts to a substance in an exaggerated or inappropriate way. The symptoms of anaphylaxis range from less to more severe, and can be life‐threatening. This Manifesto focuses on anaphylaxis resulting from allergic reactions (IgE‐mediated induced anaphylaxis). To be clear for laypeople, we use the term ‘serious allergic reactions’ instead of IgE‐mediated induced anaphylaxis. We describe high impact actions that communities can take to support people living with the risk of serious allergic reactions. Such reactions are common, cost countries millions each year and affect people's physical and mental wellbeing and productivity.^1^, ^2^, ^3^, ^4^

The European Federation of Allergy and Airways Diseases Patients' Associations (EFA) represents 46 patient organisations. It partnered with the Global Allergy and Asthma European Network (GA^2^LEN) to create this Manifesto. GA^2^LEN, is a consortium of research and clinical centres specialising in allergic disease, including 39 accredited Anaphylaxis Centres of Reference and Excellence (ANACare) which undertake anaphylaxis education, research and advocacy around the world.

This Manifesto describes practical, actionable priorities that we are advocating for over the next 5 years. We emphasise community‐based solutions that involve affected people, families, primary healthcare and schools because successful prevention and management goes beyond allergy specialists alone. We do not detail treatment such as immunotherapy for the underlying conditions. This is important, but we want to widen the conversation. The core principle is that we can achieve more together than by isolated efforts. We want to break down silos between sectors and regions to prioritise global prevention, including modifying risk factors and considering social and cultural determinants.

The Manifesto is written in an easy‐to‐read style for patients and families, people in non‐clinical roles, policymakers and healthcare workers beyond the allergy speciality. We aim to spark discussion and inspire teams to collaborate on priorities that resonate most with them.

## 2 HOW DID WE IDENTIFY PRIORITIES?

A 12‐person working group with patient organisations, allergy specialists, immunologists, paediatricians, educators, primary care, researchers and others oversaw the process to build consensus on patient‐centric advocacy priorities. We consulted directly with over 200 organisations representing thousands of stakeholders. A panel of 67 international experts reviewed and signed off the priorities.

At the beginning of the process, we extracted areas for development from recent guidelines, reviews of published evidence and primary research.^5^, ^6^, ^7^, ^8^, ^9^, ^10^, ^11^, ^12^, ^13^, ^14^ We asked 85 GA^2^LEN and EFA stakeholder organisations to identify gaps in current practice and actions that would make the greatest difference in people's lives. We had meetings and telephone calls with experts from over 30 countries. We canvassed opinions at conferences and six targeted meetings during 2023 and 2024. We involved a qualitative researcher who used grounded theory and content analysis to identify the most common priorities from all submissions.^15^, ^16^ There was significant consensus across all stakeholders, so a Delphi method was not needed to reach agreement.

The working group used a consensus process at a series of meetings to review all feedback and confirm the top 10 priorities. The draft priorities were reviewed and formally voted on by 80 people at an international conference. Final validation came from 67 organisational stakeholders including EFA, international patient organisations and GA^2^LEN organisations and members (Box 1).

**Box 1 Methods used to identify practical priorities for supporting people at risk**

1

- Submissions from 39 ANACare clinical and research centres, EFA (representing 46 patient organisations in 28 countries), 10 patient organisations and wider stakeholders.
- Literature review of gaps in diagnosis and management reported in systematic reviews, primary research studies and recent guidelines.
- Meetings and telephone calls to gain feedback from multidisciplinary and multi‐professional teams from 30+ countries, including patients and families,
- Content analysis and grounded theory approach to identify themes across all submissions, conversations and meeting notes.
- Reviewing and voting for priorities by 80 experts at 2024 GA^2^LEN ANACare Anaphylaxis and Food Allergy conference
- Oversight and final prioritisation by 12‐person expert panel working group using a consensus approach, taking into account all feedback on draft Manifesto from over 200 international stakeholders.
- Written review and validation of priorities from a panel of 67 experts.

## 3 WHAT ARE SERIOUS ALLERGIC REACTIONS?

Patient organisations said that it was important to put the priorities in context by providing a simple description of what serious allergic reactions are and why it is important to tackle them. The next sections summarise this briefly. We do not cover all details or nuances.

An allergic reaction occurs when a person's immune system mistakenly perceives a foreign substance or food as being harmful. The immune system responds by releasing chemicals to try to tackle the substance. This causes the symptoms of an allergic reaction. Serious allergic reactions are called IgE‐mediated anaphylaxis.

There are various definitions of serious allergic reactions.^17^, ^18^, ^19^, ^20^ A refined consensus definition is in press.^21^ For the purpose of this paper, we consider a serious allergic reaction to be when serious symptoms develop within minutes or hours of exposure to an allergen. The symptoms can range from what may initially seem mild (e.g. rash) to very severe. Symptoms can become more severe quickly. Very severe reactions can be life‐threatening, but many people experience much less severe symptoms. More than one body system is usually affected, including the skin, breathing system, digestive tract, and heart and blood vessels. Examples of symptoms include itchy skin, rash, hives/welts, difficulty breathing, wheezing, cough, swelling of the tongue, lips, throat or other parts of the body, difficulty swallowing, abdominal pain, nausea, vomiting, diarrhoea, changes in heart rhythm or blood pressure, flushing, and feeling faint.^22^ In other words, anaphylaxis is much broader than ‘anaphylactic shock’.

## 4 HOW COMMON ARE SERIOUS ALLERGIC REACTIONS?

Allergies are long‐term conditions. People will have periods when they manage their risk without incident and other periods of crisis when they may experience a serious allergic reaction to food, medication, venom, or other substances. Serious allergic reactions are common and affect thousands of people globally each day.

Up to 3%–5% of the population may experience a serious allergic reaction in their lifetime.^23^ This means that up to 1 in 20 people may be directly affected, plus their family and supporters.^24^ Most statistics are based on large population studies, hospital records or national registries. The figures may still underestimate the numbers due to misdiagnosis and misclassification.^25^

There may be over 5 million serious allergic reactions across the world each year.^26^, ^27^ 25%–50% of people who experience a serious allergic reaction have one or more further episodes.^28^ Serious allergic reactions are more common in children, especially those aged under 5 years, whereas fatal reactions are more common in adults, especially those aged 18–30 years.^29^, ^30^, ^31^

Serious allergic reactions are not usually fatal, but there are an estimated 8000 deaths per year across the world related to various allergens.^32^ To put this in perspective, each year about 1.2 million people die in vehicle accidents globally.^33^, ^34^

In recent decades, there has been an increase in the number of reported serious allergic reactions and associated emergency department visits and hospital admissions.^35^, ^36^, ^37^ It is unclear whether this is because reactions are becoming more common or because people are better at identifying them. Statistics from the UK, USA and Europe suggest that death due to an allergic reaction is not increasing.^38^

## 5 WHAT CAUSES SERIOUS ALLERGIC REACTIONS?

Foods, medicines and venom (such as bee and wasp stings) are the most common triggers of serious allergic reactions.^39^, ^40^, ^41^, ^42^, ^43^, ^44^ Food is the most common cause of serious allergic reactions in children. Medications and venom are the most common causes in adults.^28^, ^45^

In ‘westernised’ countries, the most common food triggers in children are peanuts, tree nuts, cow's milk and hen's eggs.^46^, ^47^ In adults, common food triggers include shellfish and wheat.^48^, ^49^ There are many other possible food and non‐food triggers, and they differ between regions.^50^, ^51^, ^52^, ^53^ Examples of other triggers include antibiotics, general anaesthetic, painkillers and latex.^35^, ^54^

## 6 WHAT IS THE IMPACT OF SERIOUS ALLERGIC REACTIONS?

Serious allergic reactions have immediate physical effects, such as difficulty breathing, vomiting and abdominal pain. Most physical symptoms only last for a short time, but can cause discomfort and anxiety for the person and their family. As previously stated, fatal reactions are rare, with 1–2 deaths per million people per year.^55^

Serious allergic reactions can impact on the quality of life of people affected and those around them.^56^, ^57^, ^58^, ^59^ People with allergies may feel anxious about having a reaction and limit their social activities and behaviours.^60^, ^61^, ^62^ They may feel stigma and avoid eating out or travelling. They may avoid talking about past experiences. People with a food allergy may avoid certain foods or buy substitutes, which can be costly and add extra preparation time.^63^ In children and people with multiple food allergies, avoiding food groups may affect nutrition.^64^ People from less advantaged socio‐economic groups may bear a higher burden because managing allergies may be more difficult or costly for them.^65^

Family members of people at risk are often upset and worried when someone has a serious allergic reaction. They may also feel anxious and protective when helping people manage their risk. They may alter their own behaviours and activities to adapt to their loved one's needs.

Serious allergic reactions also impact on health and education services. Around one quarter of affected people experience their first serious allergic reaction at school or preschool/kindergarten.^66^ Healthcare and education professionals therefore need to learn how to minimise the risk and how to identify and manage serious allergic reactions, as well as make sure emergency treatment is available.

Researchers have estimated that each developed country spends millions of currency every year on emergency department visits, hospital admissions and other direct health costs for serious allergic reactions.^67^, ^68^, ^69^, ^70^, ^71^, ^72^ These costs are increasing, in line with higher rates of serious allergic reactions.^73^ There are also economic impacts from reduced quality of life and lost productivity at work and school. Families bear around half of the cost, with the other half covered by healthcare services, insurance, employers and governments.^74^

There are also costs and challenges for the food, hospitality and travel industries. This includes the expense of identifying, labelling and segregating allergenic ingredients and complying with regulations to ensure products are safely prepared and handled.^75^

## 7 HOW ARE SERIOUS ALLERGIC REACTIONS DIAGNOSED AND MANAGED?

Many clinical guidelines and practice parameters describe ways to diagnose and manage serious allergic reactions in an emergency, as well as longer‐term management and prevention.^76^, ^77^, ^78^ Most guidelines are based on expert opinion and clinical experience because there is limited high quality research comparing treatment approaches.^79^, ^80^

The exact approaches for diagnosis and emergency management differ by region. As a general summary, a clinician will usually diagnose a serious allergic reaction based on reviewing an individual's symptoms and history, sometimes with follow up blood tests weeks later.^81^

The recommended emergency treatment during a serious allergic reaction initially includes^82^, ^83^, ^84^:

- distancing the person from the allergen where possible (e.g. removing latex or insect)
- encouraging people to lie flat with their legs raised to help circulation, or sit up if they are wheezing
- using adrenaline (also called epinephrine) as the first‐line treatment if symptoms warrant it or if in any doubt because symptoms can escalate rapidly
- using antihistamines for skin symptoms if needed

Adrenaline is usually injected into the outer upper thigh muscle, either by the person themselves, a caregiver, or a healthcare or education professional. Alternatives such as adrenaline nasal sprays are becoming available in some countries.

People are encouraged to call for emergency advice as soon as they start experiencing symptoms. In some regions, selected individuals may be advised to monitor symptoms in the community, whereas in other countries, everyone is asked to go to the emergency department, especially after using adrenaline. Most serious allergic reactions respond to one to two doses of adrenaline, but more doses may be needed. For urgent escalation, intravascular fluid replacement may be needed then intravenous adrenaline.^85^, ^86^

Guidelines are less definitive about the ‘must dos’ for the ongoing management of people at risk of serious allergic reactions. There is less research on the effectiveness of initiatives, apart from for people with food allergies. In general, studies suggest that people should have a written plan for ongoing management and emergencies based on individualised assessments of risk.^87^ They should be trained in how to avoid allergens inside and outside of the home and be prescribed approved adrenaline self‐administration devices to keep on hand.^88^ They should be supported to address other conditions such as asthma because poorly controlled asthma can increase the risk of serious allergic reactions.^89^ They should have access to accurate information about the presence of food allergens on pre‐packaged and non‐pre‐packaged food.^90^ Research also suggests considering options that may help targeted people better tolerate allergens, such as allergen‐specific immunotherapy and biological drugs.^91^, ^92^

## 8 WHAT ARE THE KEY CHALLENGES AND PRIORITIES?

A panel of other international experts previously identified *research* priorities for serious allergic reactions.^93^ The research priorities were largely clinically‐focused such as improving diagnostic criteria, identifying ways to test for anaphylaxis, standardising the criteria for admitting people to hospital and observing them, and identifying best practices in immunotherapy to help people increase tolerance to allergens. We support all of these priorities.

To add to this, GA^2^LEN, EFA and international patient organisations aimed to highlight practical actions that patient groups, educational institutions, healthcare professionals, the food and transport industries, employers and policymakers can take or campaign for to improve the lives of people with allergies. There was significant consensus on the issues raised in evidence reviews and expert submissions to our prioritisation process in 2023 and 2024.

We identified the top 10 practical priorities that experts and evidence suggested may make the greatest difference (Box 2). We focused on things that are best done collaboratively, recognising that effort is needed at the level of individuals, organisations and wider systems.

**Box 2 10 practical priorities to help prevent and manage serious allergic reactions**

1

**Table:** 

Target group	Prevention	Management
Patients and the public	1. Focus on **increasing awareness** about how to identify, respond to and proactively manage serious allergic reactions and triggers. Target groups include people with allergies (especially adolescents and young adults), their families, the public, school personnel, people working in food and hospitality industries and employers. This may include public information campaigns with social media, influencers and celebrities, games, posters, school lessons and other novel approaches. It may also include education sessions, videos, leaflets and posters for target groups and practical ways to support people who may experience anxiety and bullying as a result of their allergy	2. Empower people to help themselves by coproducing a template for a consistent easy to read/infographic **self‐management plan** for people managing allergies, including clear guidance about the symptoms of serious allergic reactions and when and how to use adrenaline
Healthcare professionals	3. **Train existing and future healthcare professionals**, including those working in primary and community care and emergency physicians, to proactively support people at risk. Training is needed to support both prevention and management, including how to identify and manage serious allergic reactions, indications for adrenaline and other treatments, the importance of prescribing and showing people when and how to use adrenaline devices, and referring to an allergy specialist and psychosocial support	4. Upskill health professionals to use **quality of life assessments** and validated risk assessments routinely, including in primary care and community settings. Empower professionals to direct people to the wealth of resources available, including community peer support groups
Wider systems	5. Implement **legal and healthcare policies** such that serious allergic reactions/anaphylaxis is categorised as a medical condition requiring proactive management. This will help people at risk maintain their right to be included and not discriminated against in all aspects of society, including education and work	7. Improve **access to and legal oversight of approved adrenaline devices**, so that patients, nurses, school staff and community workers feel confident using adrenaline quickly when other specialists are not present to oversee. This includes publicly funding and routinely prescribing two adrenaline self‐administration devices for people at risk and improving global access to adrenaline in health, education, work and appropriate public settings
	6. Implement evidence‐based **integrated care pathways** across healthcare systems so everyone at risk of a serious allergic reaction can easily access risk assessment, education, dietetic support, diagnosis, treatment and follow‐on care, including immunotherapy if appropriate. This includes investing in multidisciplinary personnel to provide support and using a dashboard of indicators to track progress over time	8. Fund easy access to an evidence‐based package of **psychoeducational interventions** to address the psychological burden of people living with allergy and their families. This may include online support, individual counselling, group sessions, apps, peer support groups, etc., with evaluation of the most effective approaches and how best to target them to different groups
Food and hospitality industry	9. **Educate the food, hospitality and travel industries** about food allergy versus intolerance, allergen control and the best ways of communicating allergens to consumers by working closely with patient groups. This may include building business cases around the financial and reputational impact if businesses do not train staff and adapt processes	10. Legislate for consistent food and hospitality industry policies to improve allergen control and communication processes. This includes considering mandatory **food allergen labelling** using clear wording constructed alongside patient groups (precautionary allergen labelling)

We recognise that clinical and legal approaches vary in different countries and regions. These 10 priorities do not seek to mandate specific interventions, devices, or wording. They call on all of us to work together on raising awareness, making sure that people have access to the support needed and agreeing appropriate approaches for prevention and management that can be adapted to local contexts.

The 10 priorities can be divided into three themes:

- Raising awareness about how to prevent and manage serious allergic reactions in a wide variety of stakeholders.
- Ensuring that people have access to and are confident using the most effective treatments during serious allergic reactions.
- Multi‐faceted approaches to support people at risk in the longer‐term.

Guidelines and studies have already identified these as ‘must do’ activities. We call on partners around the world to collaborate to turn research into practice.

### 8.1 Theme 1: Train people to recognise and manage serious allergic reactions

A new consensus definition of serious allergic reactions (anaphylaxis) is being published.^21^ However, even with agreed criteria, we still need to help people recognise and manage serious allergic reactions. These reactions may not be well recognised in primary healthcare, educational institutions, eating places or even in emergency departments.^94^, ^95^, ^96^, ^97^ Patient organisations contributing to the Manifesto said that professionals' lack of awareness of symptoms can leave patients feeling isolated, confused and as though they are not taken seriously.

For this reason, many of our top 10 priorities for action focus on raising awareness amongst the public, education professionals, healthcare professionals and the food industry. This includes improving undergraduate and postgraduate education and ongoing training about allergy and serious allergic reactions for family doctors, nurses, paediatricians, emergency care staff, people working in schools and universities, patients, families, the hospitality industry, eating places and the wider public. Standardised medical record forms with diagnostic prompts and regular anaphylaxis audits may raise awareness in healthcare settings.^98^ Primary healthcare settings and the food industry have been under‐represented in previous education or promotional campaigns.^99^ There are guidelines summarising effective strategies for schools and childcare settings, but regions need support to help implement these consistently.^100^, ^101^, ^102^

There is scope for greater collaboration among patient organisations and others, including sharing educational resources to reduce duplication. There may be a particular gap in resources tailored to young adults and adults.

### 8.2 Theme 2: Increase access to and confidence using adrenaline

Guidelines state that people should receive adrenaline as quickly as possible when a serious allergic reaction is suspected.^103^ However, many people do not receive adrenaline as the first‐line treatment or do not receive it quickly enough.^88^, ^104^ Therefore, it is a priority to increase knowledge and confidence in using adrenaline among people with allergies, their families and healthcare and education professionals. It is also a priority to ensure that adrenaline is readily available in health, education, work and appropriate public settings.^105^, ^106^, ^107^ We support using the name adrenaline consistently worldwide, either alone or simultaneously alongside the alternative name epinephrine.

Lives could potentially be saved if people at risk of serious allergic reactions carried an approved adrenaline self‐administration device and were not afraid to use it quickly if symptoms developed. However, research suggests that fear, a lack of confidence and distrust of adrenaline are barriers for people with allergies and healthcare and education professionals.^108^, ^109^, ^110^, ^111^, ^112^ Of those prescribed adrenaline autoinjectors, 25%–50% may not carry them routinely. Many say they do not feel confident using them.^113^, ^114^ Cost can be a significant barrier to prescribing and using adrenaline self‐administration devices. Public financing is needed to ensure equity of access across patient groups and regions.

Good education, repeated at regular intervals, would help to make best use of available devices, including devices like nasal sprays recently or soon to be released.^115^ In some regions people may receive a different adrenaline autoinjector from the pharmacy than they were originally prescribed, so it is important to make sure they receive training about any substituted devices. Making education fun through games and videos and involving family, friends, community groups and influencers may be part of a wider educational campaign for children and adolescents.^116^

Adrenaline may not be the first medication that healthcare professionals use when people are having a serious allergic reaction, even in the emergency department.^117^ Healthcare professionals and those in training therefore need education to understand that adrenaline is a safe and effective first‐line treatment.^118^, ^119^

Based on research, expert opinion and patient feedback, we propose that it is a priority to^120^, ^121^, ^122^, ^123^:

- publicly fund prescribing two approved adrenaline self‐administration devices at a time for each person at risk to keep with them
- make approved adrenaline devices available in kindergartens, schools, universities, workplaces and public places such as cinemas and food courts
- put legal safeguards and training in place for nurses, teachers and others who need to use adrenaline in an emergency, without specialist supervision

### 8.3 Theme 3: Invest in prevention and long‐term management

Identifying and treating people experiencing a serious allergic reaction is only part of the solution. Preventing reactions is also a priority, with actions targeting individuals, organisations/sectors and regions.

Up to half of people who experience a serious allergic reaction have a recurrence. It is essential to prescribe them adrenaline self‐administration devices and refer them to an allergy specialist to codevelop a care plan that recognises individual risks. People may also benefit from psychological support to help cope with the anxiety of being at risk or having experienced a reaction in the past.^124^

People who are allergic to food should be followed up at regular intervals by a specialist dietitian for guidance on avoidance, prevention of reactions in and outside the home, understanding precautionary allergen labels and maintaining an optimal diet.^125^ Follow up by an allergy specialist is also helpful because people may grow out of allergies or better tolerate (food) allergens over time. It is also helpful to let people know about peer support, such as online forums or in‐person support groups where patients share experiences and feel less alone.

Guidelines recommend this sort of follow‐up but it is often not implemented consistently in practice. For example, fewer than half of the people seen in the emergency department with a serious allergic reaction are prescribed adrenaline self‐administration devices for future use. Only around one third are referred to an allergy specialist.^126^ Professionals may not be aware of the range of patient support organisations available in the voluntary and community sector. This is why our top 10 priorities include developing more holistic support options, such as integrated care pathways, easy‐to‐use self‐management plans, and easier access to psychosocial support. Although this Manifesto does not delve into specific treatments for underlying conditions, we support expanding immunotherapy and biological therapy in targeted individuals to protect against serious allergic reactions after accidental allergen exposure.

In terms of wider societal change, we advocate for policies and practices that recognise serious allergic reactions as a significant medical condition, requiring adaptations and regulations so people at risk are not disadvantaged or discriminated against.

Food is the most common trigger for serious allergic reactions. The food and hospitality industries have a key role in preparing safe food, including risk assessment, quality control and informing about the presence of food allergens.^127^, ^128^ Patient groups have raised concerns that voluntary use of precautionary ‘may contain’ labels is not enough to address the presence of food allergens. These groups call for mandatory worldwide standards for labelling allergens in ingredients and potential unintended allergens in pre‐packed and non‐pre‐packed food (precautionary allergen labelling).^129^ There are data to quantify allergen reference doses for some foods, but more work is needed here.^130^

## 9 SUMMARY: HOW CAN WE IMPROVE PEOPLE'S LIVES?

GA^2^LEN and EFA call on all stakeholders to actively tackle the challenges facing people at risk of serious allergic reactions. Box 2 summarises priorities we can address first, working together across sectors and regions.^131^, ^132^ Our priorities include actions for individuals, organisations and wider systems.

Our call to action is not about reinventing the wheel. It is about highlighting areas that would make a significant difference if patient organisations, healthcare organisations, governments, researchers and the food industry collaborated to address them. What is innovative here is that numerous clinical, research and patient organisations have joined together to call for practical action. This is also a blueprint for regions with emerging allergy epidemics due to lifestyle changes and industrialisation.

Many of our priorities revolve around raising awareness. Wider awareness is essential to help reduce stigma and make it easier to accommodate food service and travel. There is limited evidence about the most effective approaches for public and professional education, so this is a significant gap for future research. Educational and support initiatives have been tried in various communities, and some good work has been done around school campaigns and self‐management plans.^133^, ^134^, ^135^, ^136^, ^137^, ^138^, ^139^, ^140^ However, given the large scale of the issue and the significant cost of serious allergic reactions for both individuals and societies, it is time for more consistent international agreements, templates and campaigns to improve people's wellbeing and save lives. Patient organisations told us that there is scope for patient organisations to work together more across geographies to share good practice, resources and ideas.

Other priorities focus on interventions at the level of healthcare systems or regulatory frameworks. These approaches require resources to implement. Costs would likely be offset by reducing the amount that countries spend caring for people suffering serious allergic reactions, especially when initiatives target those most at risk.^141^, ^142^, ^143^, ^144^, ^145^, ^146^, ^147^

Serious allergic reactions vary widely. They differ in clinical presentation, people's susceptibility and the effectiveness of responses.^148^ There is no one‐size fits all approach. Personalised strategies are needed for emergency and long‐term management. The key to developing and delivering those personalised approaches is taking action on education, care pathways and legislation (Box 3).

**Box 3 Key take home messages**

1

1. Serious allergic reactions (IgE‐mediated anaphylaxis) are common, can have a significant impact on physical and mental health and can be costly for governments, employers and families. They are more than just ‘anaphylactic shock’.
2. There are practical actions that all stakeholders can take right now or campaign for together:
  - Launch publicity campaigns about what serious allergic reactions are and how to treat them for members of the public, patients, family, food industry personnel, healthcare and education professionals and first responders.
  - Make sure that people at risk have easy access to adrenaline, including prescribing and funding approved devices; training patients and professionals about when and how to use adrenaline; and progressing laws around access to adrenaline in public places and legal safeguards for people who administer adrenaline in an emergency.
  - Provide holistic ongoing support, including self‐management plans, risk assessment, psychosocial and dietetic care, integrated care pathways and mandatory precautionary food allergen labelling for ingredients and unintended allergen presence.
3. If in doubt, use adrenaline quickly when a serious allergic reaction is suspected.

We hope that organisations will use this list of priority areas when considering what to focus on and fund. Individual organisations, collaborations, or regions will not be able to tackle every priority immediately. Groups may focus on one or two areas first, and share their experience widely to spread good practice. For example, over the next 3–5 years GA^2^LEN ANACare centres have committed to testing integrated care pathway models and creating consistent education resources for healthcare professionals, including those working in primary care. EFA organisations will contribute to these initiatives and work with partners on high profile public health campaigns for patients and the public.

## AUTHOR CONTRIBUTIONS

**Antonella Muraro**: Conceptualization; funding acquisition; methodology; validation; supervision; project administration; writing—review & editing. **Debra de Silva**: Conceptualization; investigation; writing—original draft; methodology; validation; writing—review & editing; formal analysis; data curation; project administration. **Marcia Podesta**: Conceptualization; supervision; writing—review & editing. **Aikaterini Anagnostou**: Writing—review & editing; conceptualization. **Victoria Cardona**: Conceptualization; writing—review & editing. **Susanne Halken**: Conceptualization; writing—review & editing. **Pete Smith**: Conceptualization; writing—review & editing. **Luciana Kase Tanno**: Conceptualization; writing—review & editing. **Paul Turner**: Conceptualization; writing—review & editing. **Margitta Worm**: Conceptualization; writing—review & editing. **Montserrat Alvaro‐Lozano**: Conceptualization; writing—review & editing. **Stefania Arasi**: Writing—review & editing. **Anna Asarnoj**: Writing—review & editing. **Simona Barni**: Writing—review & editing. **Kirsten Beyer**: Writing—review & editing. **Lucy A. Bilaver**: Writing—review & editing. **Andrew Bird**: Writing—review & editing. **Roberta Bonaguro**: Writing—review & editing. **Helen A. Brough**: Writing—review & editing. **R. Sharon Chinthrajah**: Writing—review & editing. **Emma E. Cook**: Writing—review & editing. **Céline Demoulin**: Writing—review & editing. **Antoine Deschildre**: Writing—review & editing. **Timothy E. Dribin**: Writing—review & editing. **Motohiro Ebisawa**: Writing—review & editing. **Montserrat Fernandez‐Rivas**: Writing—review & editing. **Alessandro Fiocchi**: Writing—review & editing. **David M. Fleischer**: Writing—review & editing. **Eleanor Garrow**: Writing—review & editing. **Jennifer Gerdts**: Writing—review & editing. **Mattia Giovannini**: Writing—review & editing. **Kirsi M. Järvinen**: Writing—review & editing. **Mary Kelly**: Writing—review & editing. **Edward F. Knol**: Writing—review & editing. **Gideon Lack**: Writing—review & editing. **Francesca Lazzarotto**: Writing—review & editing. **Thuy‐My Le**: Writing—review & editing. **Stephanie Leonard**: Writing—review & editing. **Jay Lieberman**: Writing—review & editing. **Michael Makris**: Writing—review & editing. **Lianne Mandelbaum**: Writing—review & editing. **Mary Jane Marchisotto**: Writing—review & editing. **Gustavo Andres Marino**: Writing—review & editing. **Francesca Mori**: Writing—review & editing. **Caroline Nilsson**: Writing—review & editing. **Anna Nowak‐Wegrzyn**: Writing—review & editing. **Mikaela Odemyr**: Writing—review & editing. **H. N. G. Oude Elberink**: Writing—review & editing. **Kati Palosuo**: Writing—review & editing. **Nandinee Patel**: Writing—review & editing. **Jennifer Pier**: Writing—review & editing. **Sung Poblete**: Writing—review & editing. **Rima Rachid**: Writing—review & editing. **Pablo Rodríguez del Río**: Writing—review & editing. **Maria Said**: Writing—review & editing. **Hugh A. Sampson**: Writing—review & editing. **Angel Sánchez Sanz**: Writing—review & editing. **Sabine Schnadt**: Writing—review & editing. **Fallon Schultz**: Validation. **Alice Toniolo**: Writing—review & editing. **Julia E. M. Upton**: Writing—review & editing. **Carina Venter**: Writing—review & editing. **Brian P. Vickery**: Writing—review & editing. **Berber Vlieg‐Boerstra**: Writing—review & editing. **Julie Wang**: Writing—review & editing. **Torsten Zuberbier**: Supervision. **Graham Roberts**: Conceptualization; writing—review & editing.

## CONFLICT OF INTEREST STATEMENT

A separate file has been provided listing all potential interests of the authors.

## Data Availability

Data sharing not applicable to this article as no datasets were generated or analysed during the current study.
